# Effect of unilateral pulsed jet lavage prior to vertebroplasty on the intravertebral pressure and cement distribution

**DOI:** 10.1186/s13018-020-01779-3

**Published:** 2020-07-11

**Authors:** Jun Yan, Qiaohui Liu, Yanping Zheng, Ziqun Liu, Xinyu Liu, Xun Guo, Penghui Liu, Peng Chen, Suomao Yuan, Yonghao Tian, Wanlong Xu

**Affiliations:** 1 Department of Orthopedics, Qilu Hospital, Shandong University, Jinan, Shandong People’s Republic of China; 2 Department of Spine Surgery, Second Xiangya Hospital, Central South University, Changsha, Hunan People’s Republic of China; 3 Department of Pediatric Surgery, The Second Affiliated Hospital of Xi’an Jiaotong University, Xi’an, Shanxi People’s Republic of China

**Keywords:** Percutaneous vertebroplasty, Unilateral pulsed jet lavage, Intravertebral pressure, Cement distribution

## Abstract

**Background:**

Percutaneous vertebroplasty is the most common treatment for osteoporotic vertebral compression fracture. However, the morbidity of vertebroplasty-related complications, such as cement leakage, remains high. We tested a new technique of unilateral pulsed jet lavage and investigated its effect on the intravertebral pressure and bone cement distribution.

**Methods:**

Thirty lumbar vertebrae (L1-L5) from six cadaver spines were randomly allocated into two groups (with and without irrigation). Prior to vertebroplasty, pulsed jet lavage was performed through one side of the pedicle by using a novel cannula with two concentric conduits to remove the fat and bone marrow of the vertebral bodies in the group with irrigation. The control group was not irrigated. Then, standardized vertebroplasty was performed in the vertebral bodies in both groups. Changes in the intravertebral pressure during injection were recorded. Computed tomography (CT) was performed to observe the cement distribution and extravasations, and the cement mass volume (CMV) was calculated.

**Results:**

During cement injection, the average maximum intravertebral pressure of the unirrigated group was higher than that of the irrigated group (4.92 kPa versus 2.22 kPa, *P* < 0.05). CT scans showed a more homogeneous cement distribution with less CMV (3832 mm^3^ vs. 4344 mm^3^, *P* < 0.05) and less leakage rate (6.7% vs. 46.7%, *P* < 0.05) in the irrigated group than in the control group.

**Conclusions:**

Unilateral pulsed jet lavage can reduce intravertebral pressure and lower the incidence of cement leakage during vertebroplasty. An enhanced bone cement distribution can also be achieved through this lavage system.

## Background

Percutaneous vertebroplasty (PVP) or percutaneous kyphoplasty (PKP) is effective in the treatment of osteoporotic vertebral compression fractures. These techniques can provide instant pain relief [1–3] and stabilize vertebral fracture [3–5]. However, leakage during PVP or PKP remains a concern among surgeons. Some studies have shown that intravertebral lavage prior to cement injection can effectively remove the vertebral fat and bone marrow and reduce the morbidity of extravasation [6, 7]. Previously reported lavage methods involved bilateral operation [8–10], but the unilateral pulsed jet lavage method has not been described. Thus, we designed and used a unilateral pulsed jet lavage system to investigate the effect of unilateral irrigation prior to vertebroplasty on leakage, intravertebral pressure, and cement distribution.

## Methods

Thirty intact cadaver lumbar vertebrae (L1-L5) were harvested from ten human cadaveric spines without a known history of spinal pathology. The age of donors was 71.5 ± 4.9 years (age range, 63–79 years). The periosteum and adjacent discs of the vertebral bodies were intact. The bone density of the vertebrae was assessed by dual-energy X-ray absorptiometry. These specimens were stored at − 20 °C. Before the experiment was conducted, the specimens were thawed at room temperature overnight.

**Fig. 1:**
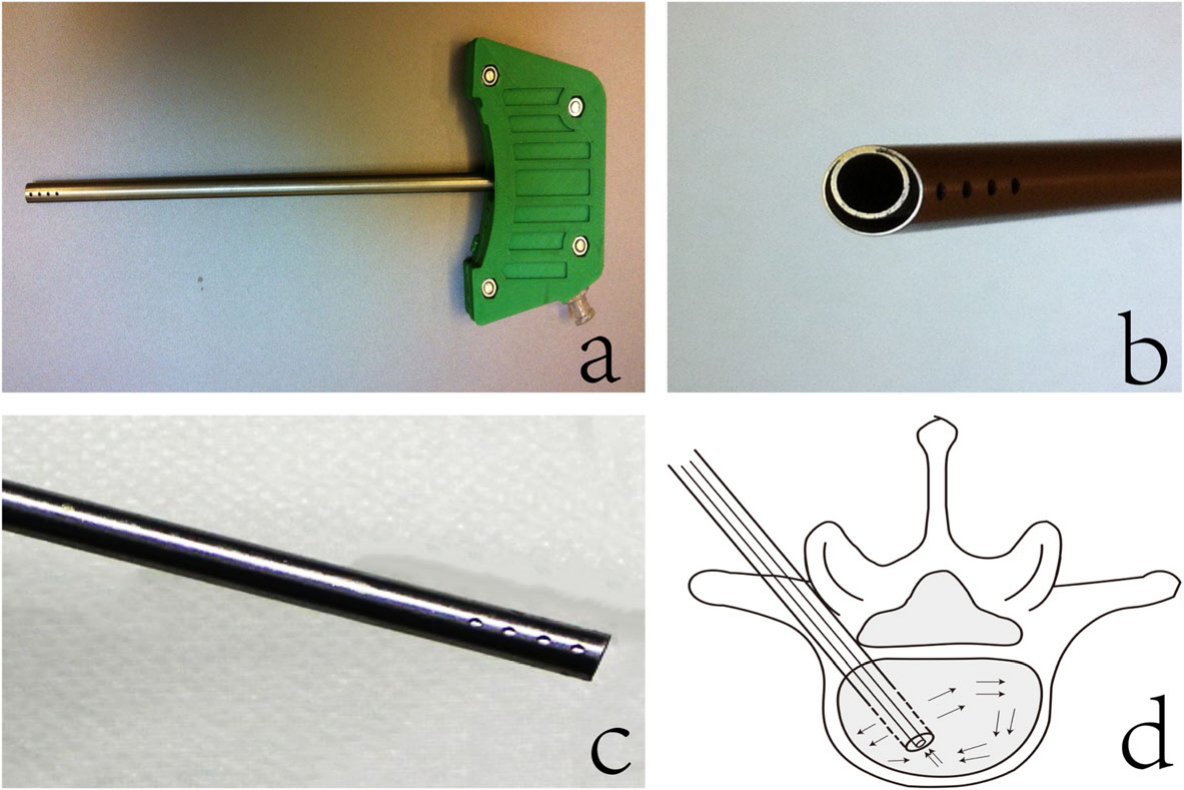
The irrigation device. **a** Full view of the device. **b** The outer conduit and inner conduit. **c** The holes on the tip of the outer conduit allowed saline to flow in different directions. **d** Saline used for lavage flowed through the holes of the outer conduit and went around in the vertebral body. Meanwhile, the inner conduit created negative pressure to regain the saline. Arrows indicated the flow of water

The vertebrae were allocated randomly into two groups (namely, with and without irrigation) with 15 samples per group. Then, an 11-gauge bone biopsy needle (SterilabHiggins KB S.r.l, Buccinasco, Italy) was inserted through the right pedicle of each vertebra, and an irrigation device was installed via the channel made by the biopsy needle. The irrigation device was a novel cannula with two concentric conduits. The outer conduit was used for the pulsed jet lavage, and the inner conduit was used to regain saline (Fig. 1). Saline (200 mL) was pulsed to each vertebra, and regaining started simultaneously. Saline with the vertebral fat and marrow was sucked out completely from the vertebral bodies through the device. No operation was performed on the control group in this step. Before and after irrigation, a miniature video camera system was installed in the vertebral body to assess the lavage effect. Prior to the cement injection, a digital manometer (GM511, Shenzhen Jumaoyuan Science and Technology Co., Ltd., Shenzhen) was placed just beyond the left pedicle-body junction of each vertebra in both groups to monitor and record the intravertebral pressure during cement injection. A 1.5-mL injection cannula was used to inject radio-opaque polymethylmethacrylate bone cement (Simplex P, Stryker Howmedica Osteonics, Mahwah, NJ, USA) twice into each vertebra at a rate of 3 mL/min in accordance with standard vertebroplasty. The bone cement was mixed in accordance with the manufacturer’s instructions (20 mL of monomer to 40 g of powder). Afterward, 3 mL of bone cement was injected into each vertebral body. Computed tomography (CT) was performed to estimate the distribution of cement in the vertebral body and calculate the CMV. The cement mass edge was drawn in each cross-sectional CT image by using the CT software (Mimics Innovation Suite 21), and the area of the marked region was calculated. Then, CMV was obtained by multiplying the cement area with the height of each cross-section.

The statistical outcomes of the CMV and intravertebral pressure were presented as mean ± standard deviation. A Mann-Whitney *U* test was used to analyze the differences in the volume and pressure between the two groups. Fisher’s exact test was conducted to analyze the leakage rate. *P* ≤ 0.05 was considered statistically significant. Statistical analysis was carried out by using SPSS 26.0.

## Results

**Fig. 2:**
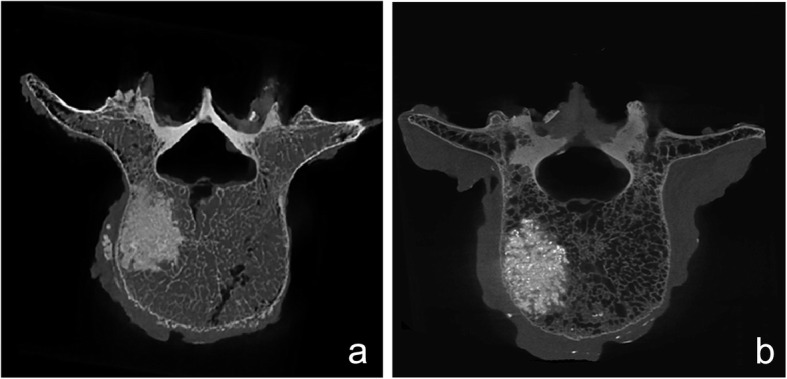
CT scans of vertebrae after vertebroplasty. **a** CT scans showed that the cement mass in the vertebra with irrigation was homogeneous. **b** The cement mass was more irregular in the unirrigated specimen than in the irrigated vertebra

**Fig. 3:**
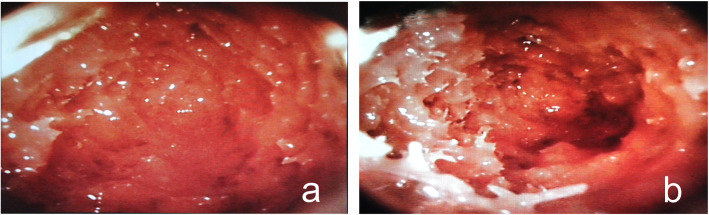
The images gained from the video camera. **a** Before pulsed jet lavage, the vertebral body was filled up with fat and marrow. **b** After lavage, the intravertebral fat and marrow were removed and the trabecula was recognizable

The main outcome measures are listed in Table 1. There is no significant difference in bone density between the two groups. Some images from a video camera and CT scans are displayed in Figs. 2 and 3.

**Table 1:** Summarized data of the results

	Irrigated group	Unirrigated group	*P* value
Bone density (g/cm^3^)	0.691 ± 0.136	0.672 ± 0.118	0.713
Cement extravasation	6.7% (1 of 15)	46.7% (7of 15)	0.035
Cement mass volume (mm^3^)	3832 ± 596	4344 ± 653	0.041
MP (kPa)	2.22 ± 0.37	4.92 ± 2.87	0.001*
HP (kPa)	1.43 ± 0.31	3.97 ± 2.01	0.001*

*MP* maximum intravertebral pressure, *HP* half volume pressure

*Significant difference

### Leakage rate

Cement leakage was evaluated on the basis of the CT images. Cement leakage occurred in one (6.7%) of the irrigated specimens and in seven (46.7%) of the unirrigated ones (Table 1).

### Cement distribution

CT images showed the distinct distributions of cement in the two groups. The bone cement mass was homogeneous and had more uniform density in the irrigated group than in the unirrigated group. The cement distribution of the unirrigated vertebrae was more irregular than that of the irrigated group (Fig. 2).

### CMV

The Mann-Whitney *U* test results revealed smaller CMV in the irrigated group than in the unirrigated group (*P* < 0.01). The irrigated mass volume was 3832 ± 596 mm^3^, whereas the unirrigated volume was 4344 ± 653 mm^3^.

### Images from the miniature video camera system

The images from the miniature video camera system showed distinct changes in the trabeculae. Before lavage, the space of the body was filled with fat and marrow. After irrigation, these components were reduced dramatically, and the space was emptied among the trabecula (Fig. 3).

### Intravertebral pressure

**Fig. 4:**
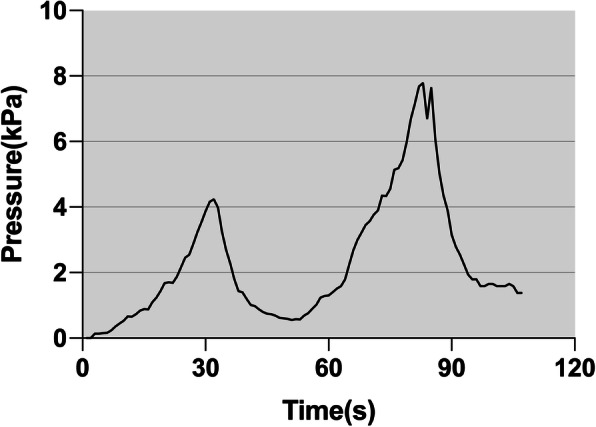
The pressure-time curves during cement injection. On the pressure curve, there were two peaks, associated with the injections of cement. Each peak pressure was produced by 1.5-ml cement. The second peak was always higher than the first one

The pressure-time curves in both groups presented two peaks (Fig. 4). A peak indicated the intravertebral pressure produced by 1.5 mL of cement. The maximum pressure of the whole injection process was equal to the top value of the second peak. The average maximum intravertebral pressure in the unirrigated group was almost twice as much as that in the irrigated group. Moreover, the increasing rate of pressure during the first fillings in the unirrigated specimens was more pronounced than that in the irrigated specimens. The half cement volume pressure (the top value of the first peak) of the unirrigated vertebrae was higher than that of the irrigated bodies (*P* < 0.01). The intravertebral pressure in the first filling was 81% of the maximum pressure in the unirrigated vertebrae, but 64% in the irrigated vertebrae.

## Discussion

The intravertebral pressure and cement distribution in cadaveric vertebroplasty with and without unilateral pulsed jet lavage were recorded and compared. The unilateral lavage method in this study was an original design applied to vertebroplasty. Many reports have shown that lavage technique can remove fat and bone marrow and reduce intravertebral pressure in vertebroplasty compared with those of conventional cement injection [6, 7, 10]. However, these trials were bipedicular, which meant that lavage was performed on one side and suction was conducted from the contralateral side. However, in clinical practice, many surgeons, including our group, still prefer unilateral vertebroplasty because this process has been reported to present short- and long-term clinical outcomes similar to those of the bilateral technique [11, 12]. The unilateral method involves shorter operation time [11, 13] and less X-ray exposure frequency and trauma [14, 15]. To our knowledge, our study was the first to report the application of unilateral lavage in vertebroplasty.

The results showed that unilateral lavage significantly reduced intravertebral pressure and cement leakage. Pulsed saline lavage could remove the fat and marrow, as shown by the intravertebral images from the miniature video camera. After irrigation, the space became clearer, and the trabeculae could be distinguished. Yang et al. [16] also reported the effect of pulsed lavage to remove the bone marrow, and similar positive outcomes were achieved. With less fat and marrow, the space in the trabeculae was expanded and would accommodate more bone cement [17–19]. Hence, less pressure would be transmitted and recorded from the contralateral side of the vertebral body. Less bone leakage could also be achieved, and this finding was reported in previous bilateral lavage studies [6, 7].

The results also showed an interesting pattern of the changes in the recorded pressure during vertebroplasty. Two peaks, which corresponded to the two steps of bone cement injection, appeared in the pressure curve. Given the accumulating effect of the bone cement, the second peak did not start from the baseline, and the value was higher than the first peak. This adding-on phenomenon showed that more elaborative inspection should be performed during the latter stage of bone cement injection to rule out cement leakage or other detrimental effects caused by higher intravertebral pressure.

CT scans were used to assess cement distribution. From gross inspection, the cement in the irrigated samples was more even and homogeneous than that in the unirrigated ones. Compared with the control group, the density of bone cement in the irrigation group tended to be more consistent and the borders were smoother. This may be a result of removing the bone marrow and fat which were barriers in the cement diffusion process. The composition of bone cement clumps was simple, and the resistance of bone cement to diffuse was reduced. So the cement diffusion tended to be regular. The leakage rate was lower in the irrigated group than in the unirrigated ones. The CMV was smaller in the former than in the latter. The CMV represented the volume, which included the filled cement, trabecula, and remaining fat and marrow. The decrease in CMV further verified the effect of removing the fat and marrow from the vertebral body through irrigation.

In this study, an originally designed unilateral pulsed jet lavage system was applied and could reduce the intravertebral pressure and leakage during vertebroplasty. One limitation of our study was its in vitro nature. Without in vivo studies, the decreased intravertebral pressure could not be directly correlated with the reduced rate of fat or cement embolism during vertebroplasty. Another limitation was the small sample size, which could cause an adverse effect on data analysis. Further in vivo studies are recommended to validate the clinical values of a unilateral jet lavage system in vertebroplasty.

## Conclusions

Unilateral pulsed jet lavage prior to vertebroplasty shows potential for clinical application. This process can reduce the cement leakage rate and improve cement distribution by removing the intravertebral fat and marrow and decreasing the intravertebral pressure.

## Data Availability

The datasets used and/or analyzed during the current study are available from the corresponding author on reasonable request.

## Abbreviations

CT: Computed tomography
CMV: Cement mass volume
PVP: Percutaneous vertebroplasty
PKP: Percutaneous kyphoplasty
HP: Half volume pressure
MP: Maximum intravertebral pressure
